# Measuring Skin Carotenoids Using Reflection Spectroscopy in a Low-Income School Setting

**DOI:** 10.3390/nu13113796

**Published:** 2021-10-26

**Authors:** Anna M. Jones, Angie Keihner, MaryAnn Mills, Barbara MkNelly, Kamaljeet K. Khaira, Jona Pressman, Rachel E. Scherr

**Affiliations:** 1 CalFresh Healthy Living, University of California, Davis, CA 95618, USA; anajones@ucdavis.edu (A.M.J.); akeihner@ucdavis.edu (A.K.); mamills@ucdavis.edu (M.M.); bmknelly@ucdavis.edu (B.M.); kjkhaira@ucdavis.edu (K.K.K.); 2 Department of Nutrition, University of California, Davis, CA 95616, USA; 3 Center for Nutrition in Schools, Department of Nutrition, University of California, Davis, CA 95616, USA; 4 University of California Cooperative Extension Butte County, Oroville, CA 95965, USA; jmpressman@ucanr.edu

**Keywords:** Veggie Meter^®^, dietary assessment, nutrition knowledge, low-income populations

## Abstract

Dietary behavior change is difficult to accurately measure in a low-income youth population. Objective tools to measure fruit and vegetable consumption without relying on self-report present the opportunity to do this with less respondent burden and bias. A promising tool for quantifying fruit and vegetable consumption via proxy is skin carotenoids as measured by reflection spectroscopy through a device called the Veggie Meter^®^. To assess whether the Veggie Meter^®^ is able to detect changes in skin carotenoids as a proxy for fruit and vegetable consumption in a low-income school setting, skin carotenoid measurements were collected at three time points, along with student level demographics, anthropometric measurements, and nutrition knowledge. A secondary goal of this study was to refine the protocol to be used based on researcher observations. Repeated measures analysis of variance with Bonferroni correction for multiple comparisons indicate that there was a significant difference in VM scores over the course of the study (F(2, 68) = 6.63, *p* = 0.002), with an increase in skin carotenoids from Fall 2018 to Spring 2019 (*p* = 0.005). This increase was sustained over the summer months when measured in Fall 2019. Changes to the protocol included the addition of a hand cleaning step and using the non-dominant ring finger for data collection. With these refinements, the results demonstrate that the Veggie Meter^®^ is usable as a non-invasive tool for measuring fruit and vegetable consumption in a population that is traditionally difficult to assess.

## 1. Introduction

The causes of childhood obesity are multifaceted with poor dietary intake remaining a key contributor. Over the past 20 years, overweight and obesity in children has risen at an alarming rate [1]. Based on serial NHANES surveys from 1999 to 2016, the estimated overall diet quality of US youth showed modest improvement, but more than half of youth still had poor-quality diets [2,3].

As youth age, the discrepancies between actual intake and recommendations continue to broaden [2,4]. One area in which all age groups fail to meet recommendations is the consumption of fruits and vegetables [5]. A recent data brief released by the National Center for Health statistics reported that only 75.3% of children and adolescents between the ages of 2 and 19 consumed any fruit (whole or 100% fruit juice) on a given day [5]. While over 90% of children and adolescents consumed vegetables on a given day, nearly half (47.5%) consumed starchy vegetables, while a much lower proportion (16.7%) reported consuming dark green vegetables. Although dietary patterns of children improved between 1999 and 2016, as measured by the American Heart Association 2020 continuous diet score, less than 1% are consuming diets that adhere to recommendations, and over half (56.1%) are consuming poor diets [2]. In addition, certain demographic groups are at higher risk for poor dietary patterns, including youth in households with incomes below 130% of the federal poverty level, parents with lower educational attainment, and/or lower food security [2]. Existing literature examining the relationship between youth food security and diet quality shows youth categorized as food insecure had significantly poorer diets, as assessed by Healthy Eating Index-2015 scores when controlling for sociodemographic characteristics [6]. Because of these subtle but substantial differences, accurately assessing dietary patterns is critical to be able to identify areas of improvement and design effective interventions for these higher risk population subgroups.

The Supplemental Nutrition Assistance Program Education (SNAP-Ed) program provides nutrition, healthy living, and physical activity education to low-income individuals and families. In the 59 years that the SNAP-Ed program has been in existence, there have been numerous studies published demonstrating the program’s efficacy and effectiveness, including improvements to diets of youth and their families in California and elsewhere [7,8].

In order to unify evaluation measures that showcase the effectiveness of SNAP-Ed nationally, an evaluation framework based on the Socioecological Model (SEM) to align evaluation with specific outcome indicators at each level of influence and within short-, medium-, and long-term time frames has been developed [9]. This comprehensive evaluation approach allows for a more complete picture of an individual’s response to a given intervention or program.

When focusing specifically on the middle childhood age group, designing and implementing multicomponent, comprehensive programs with the school as a hub have been highlighted as the most promising approach to mitigating the impacts of childhood obesity [10,11,12]. Although preventing childhood obesity as measured by BMI percentile is important, many children with BMIs in the normal range also benefit from improved dietary patterns in childhood, as these can establish lifelong habits [13,14]. Accurately measuring youth dietary behaviors to evaluate the impact of school-based nutrition interventions is notoriously difficult [15]. One method that is frequently used for collecting dietary intake data in youth are food frequency questionnaires [16]. While these tools can collect useful dietary intake data, they are not without limitations. One of these limitations is challenges with recall, especially with youth who may have more difficulty in accurately estimating the frequency with which they consume foods, particularly over longer time frames [17]. In addition, research suggests there may be social desirability bias, which can result in overreporting consumption of foods considered healthy and underreporting consumption of foods considered unhealthy, and disproportionately impacts those receiving nutrition-related programming in relation to comparison groups [18].

While these tools have drawbacks, they demonstrate that youth dietary patterns fall short of recommended intakes in every age group [2,5,19]. Being able to detect intermediate dietary change requires tools that are both objective and responsive to changes in intake [20,21,22,23].

With these considerations in mind, reflection spectroscopy has emerged as a relatively low-cost, non-invasive, and most-importantly, objective method of detecting changes in dietary behavior, specifically consumption of carotenoid-containing fruits and vegetables [24,25,26,27]. When consumed, carotenoids are deposited in the skin; greater consumption of fruits and vegetables is associated with higher amounts of detectable skin carotenoids [28]. Because of the success of using Resonance Raman Spectroscopy to measure carotenoids that are deposited in the skin, reflection spectroscopy (RS) has also shown to be valid in several age groups and highly responsive to changes in intake [29]. Specifically, the Veggie Meter^®^ (Longevity Link Corp., Salt Lake City, UT, USA) uses RS to detect carotenoids in the skin and is a non-invasive, objective indicator of approximately 30 days of fruit and vegetable intake [30]. An individual measurement takes less than 60 s using an LED lightbulb and the device itself is portable and can be set up at the school site with ease. Although a promising approach to measuring carotenoids in the skin, the Veggie Meter^®^ was relatively new technology at the time of the study. As a result, a standardized protocol for use of the Veggie Meter^®^ in schools had yet to be established.

The current study sought to determine whether the Veggie Meter^®^ is able to assess changes in fruit and vegetable consumption within an established comprehensive, school-based intervention in a low-income school [20,31,32,33,34]. A secondary goal of this study was to identify gaps in the existing protocol and refine the protocol to be used in low-income schools, streamlining the collection of data for real world program delivery at schools rather than in research settings.

The context for this study was to assess the aforementioned goal using the Shaping Healthy Choices Program (SHCP) as a backdrop. This program has been consistently implemented since 2012 and has resulted in improvements to health and diet-related outcomes [20,31,32,33,34,35,36,37]. By piloting the Veggie Meter^®^ within a well-established program with consistently positive outcomes, the protocol could be refined for use in schools.

Further, when the study was initiated, the Veggie Meter^®^ device was accompanied by a protocol developed by the manufacturer. While this protocol outlined the basic steps for usage, it did not take into account the logistics of implementing the device in school settings, in which additional steps may be required to ensure data collection proceeds smoothly. As this was the first time the device was used by this research group, it became evident that it would be necessary to supplement the Veggie Meter^®^ manufacturer protocol to render it feasible to administer in schools efficiently and minimize the amount of classroom time diverted for data collection.

## 2. Materials and Methods

A convenience sample of six third-grade classrooms in two SNAP Ed-qualifying schools (greater than 50% of students qualifying for Free or Reduced-Price meals) in Northern California were recruited to participate. In order to participate, classroom teachers for all three grade levels (3rd, 4th, and 5th) agreed to allow trained CFHL, UCCE educators to deliver the curricula across three years. Programming began in school year 2017–2018 when all participating students were in third grade.

Data collection took place before programming began (Fall 2017) and each fall and spring for the following years of the study, with the exception of Spring 2020, which was disrupted by the novel coronavirus disease 2019 (COVID-19) pandemic.

Consent packets for the larger study were distributed at two schools in English and Spanish in fall of 2017; parents returned signed consent forms to classroom teachers. Youth participating in the study were read a youth assent statement before data collection, during which they could verbally opt out. In the fall of 2018, new consent packets were distributed in one of the two schools that included informed consent to use of the Veggie Meter^®^ in addition to assessments that were previously conducted.

As part of the consent packets, parents were asked to complete a demographic questionnaire, with questions about race/ethnicity, household income, parental education, and whether there was someone who smoked tobacco within the household. In addition, parents were asked to indicate the primary and secondary caregiver, with options provided for mother, father, grandparent, and other to allow for non-traditional family structures.

### 2.1. Nutrition Knowledge

In order to establish consistency with prior implementations of the SHCP nutrition knowledge assessments were conducted. Nutrition knowledge was assessed at all timepoints using a subset of 20-items was adapted from a knowledge questionnaire developed and validated by Morris and Zidenberg-Cherr [38]. This adaptation was implemented to reduce participant burden. These data were collected for consistency with previous implementation of the SHCP.

### 2.2. Anthropometrics

Due to an established correlation between BMI and carotenoids [39], height and weight data were collected at all time points from consented and assented youth and used to calculate body mass index (BMI) percentile-for-age. Centers for Disease Control and Prevention guidelines were followed [40,41]. Briefly, height was measured to the nearest 0.1 cm using a stadiometer (Seca, Chino, CA, USA) and body weight to the nearest 0.1 kg using a portable electronic scale (Seca, Chino, CA, USA). Measurements were taken twice, in the event of more than a 0.3 cm difference in height or 0.1 kg difference in weight between measures, a third measurement was conducted. Along with date of birth, these values were used to calculate BMI percentile-for-age [20].

### 2.3. Skin Carotenoids

Skin carotenoids were assessed using the Veggie Meter^®^ beginning in Fall 2018. Single measurements were used at all timepoints to generate a Veggie Meter^®^ (VM) score. As one major outcome of this study was determination of proper protocol, procedures evolved. At all timepoints, the device was calibrated before use and after 1–2 h of continuous use or when it was moved.

As one of the aims of the study was to refine the Veggie Meter protocol for use in low-income schools, observations were made by researchers as to refinements that may improve data collection to streamline the process or align with other research being conducted. Study researchers that were present for data collection debriefed after each session to discuss challenges and possible solutions, with refinements made to the protocol based on these discussions.

### 2.4. Data Analyses

Means and standard deviations were calculated for continuous variables (nutrition knowledge, BMI percentile-for-age, and VM score). Repeated measures Analysis of Variance (ANOVA) with Bonferroni correction for multiple comparisons was used to assess changes in nutrition knowledge, BMI percentile-for-age, and VM score over time. Pearson’s correlation was calculated between change in BMI percentile and change in the VM score as well as between nutrition knowledge and VM score at each timepoint to determine if these were related. Only students with data at all timepoints were included in each respective analysis.

All quantitative analyses were completed using SPSS 26.0 and 27.0 (IBM Corp., Armonk, NY, USA). Significance was set at *p* < 0.05.

## 3. Results

In total, 35 students provided consent and assent and completed assessments for anthropometrics and VM score and were included in this sub-analysis. The majority of participants were 9 years of age at the Fall 2018 timepoint, with just over half (51.4%) female (Table 1). Students were primarily Caucasian and not of Hispanic origin (34.4%) or Latino/Hispanic (22.9%). Household income varied, with several families reporting an income below $40,000 or above $100,000. Parent or caregiver educational attainment ranged from less than 8th grade to post-graduate with the majority having completed some college. Most (82.9%) households reported not having a smoker.

Although 35 students were included in the overall sub-analysis, a total of 25 students had nutrition knowledge data for all three time points and were included in the ANOVA in Table 2. Nutrition knowledge differed significantly over the course of the study (F(2, 48) = 5.51, *p* = 0.007; Table 2), with post-hoc analysis determining there was a significant increase in knowledge between Fall 2018 and Fall 2019 (*p* = 0.006; Table 2). There were no significant differences between other timepoints. No statistically significant difference in BMI percentile was observed over all three timepoints (F(2, 68) = 2.137, *p* = 0.126; Table 2). The VM scores also significantly differed between time points (F(2, 68) = 6.63, *p* = 0.002; Table 2). Post-hoc tests showed significant increases in the VM scores between Fall 2018 and Spring 2019 (*p* = 0.005; Table 2) and between Fall 2018 and Fall 2019 (*p* < 0.05).

No correlation was found between change in BMI percentile and VM score change between Fall 2018 and Spring 2019 (r = 0.157; *p* = 0.354), or between Spring 2019 and Fall 2019 (r = 0.014; *p* = 0.930), suggesting that BMI percentile and VM score were not related among these students (Table 3). Nutrition knowledge was found to be positively correlated to VM score in Fall 2018 (r = 0.269, *p* = 0.034), but not in Spring 2019 (r = −0.018, *p* = 0.912) or Fall 2019 (r = 0.068, *p* = 0.514) timepoints, nor when the three scores were averaged (r = 236, *p* = 0.256; Table 3).

Table 4 describes refinements to the protocol made due to observations completed during the data collection processes. Observations made by researchers in Fall 2018 that led to changes included the presence of colored ink on the hands of some students as well as the tendency of students to attempt to get closer to the Veggie Meter while waiting for their turn. When assessments were conducted in Spring and Fall 2019, students cleaned hands with hand sanitizing wipes prior to Veggie Meter^®^ measurements. In addition, a line created on the ground with removable tape was added to discourage students from attempting to crowd around the device while they waited their turn. The dominant hand index finger was used with all participants during the Fall 2018 and Spring 2019 timepoints. Subsequent to this timepoint, an international meeting of Veggie Meter^®^ researchers convened and it was determined that the non-dominant ring finger should be used [42], and in Fall 2019 the non-dominant ring finger was used instead of the dominant hand index finger.

## 4. Discussion

In this study, the Veggie Meter^®^ was successfully employed in a comprehensive, school-based intervention in a low-income school focused on improving fruit and vegetable intake. Further, VM scores increased over the course of the school year (between Fall 2018 and Spring 2019) and were stable over the summer months, as indicated by no significant changes between Spring 2019 and Fall 2019. This suggests that the Veggie Meter^®^ is sensitive to changes in intake that occur as a result of school-based nutrition education. While dietary intake was only assessed by proxy using the Veggie Meter^®^, this was by design, as the Veggie Meter^®^ has been previously validated against several different criteria [43], and it was critical to determine if it can be successfully implemented in low-income schools despite the logistical challenges that accompany working in this setting. Overall, this study supports the growing body of literature that suggests that the Veggie Meter^®^ is an appropriate tool for measuring change in fruit and vegetable consumption by proxy in the school setting, especially in a low-income school setting [24,25,26].

Many of the tools used to assess dietary behaviors among youth, such as 24-h recall and food frequency questionnaires, rely on recall, which is subject to memory, social desirability bias, and respondent burden [18,44,45,46]. An issue that may be encountered in low-income populations is low response rate for lengthy food frequency questionnaires, speculated to be due to the time involved and complexity of the forms, causing insurmountable burden for youth and their parents [47]. An unexpected benefit of the Veggie Meter^®^ was that researchers noted youth, as well as classroom teachers in some cases, were very enthusiastic and engaged when the Veggie Meter^®^ was introduced, which may have increased participation. While this observation is anecdotal, a similar phenomenon was noted with wearable activity monitors that displayed user feedback in response to physical activity, which may have resulted in increased activity during the assessment period independent of the intervention [34].

With respect to the SNAP-Ed Evaluation Framework, the objective, non-invasive technique of assessing skin carotenoids with the Veggie Meter^®^ is an ideal method for evaluation that aligns with indicators of interest at the individual level, including medium-term behavioral changes in healthy eating (a SNAP-Ed Priority Outcome Indicator) and long-term maintenance of behavioral changes in healthy eating [48]. As the data collection tools recommended to assess outcome measures related to these indicators are primarily recall survey tools, the addition of skin carotenoid assessment may be of benefit. A recent study of 124 SNAP-Ed State Implementing Agencies (SIA) representing all 50 states and the District of Columbia, in which 95% of SIAs surveyed intended to impact healthy eating behavioral changes in the medium-term, further supports this [49]. As SNAP-Ed is a large nationwide program, with a budget of over $431 million in Fiscal Year 2021 [50], the ability to evaluate the effectiveness of programs funded by SNAP-Ed is essential to ensuring that these funds are directed toward interventions with measurable impact. This study demonstrating the usability of the Veggie Meter^®^ in a low-income school setting is especially relevant as schools are a key setting targeted by SNAP-Ed.

Consistent with previous SHCP implementation, nutrition knowledge increased over the course of the study [20,35,36,37]. This demonstrates alignment of the current implementation with previously published SHCP research, allowing inferences to be made about the ability of the Veggie Meter^®^ to detect changes in behavior. Changes in knowledge are thought to precede changes in behavior, as posited in the SCT, which provides the basis for many behavioral interventions [51]. However, while nutrition knowledge was found to have increased over the course of the study, an unexpected finding was that the correlation between nutrition knowledge and VM score did not persist over time; this can possibly be attributed to differences in completion of assessments, as only 25 participants had nutrition knowledge data for all three timepoints compared to 35 completing all three Veggie Meter^®^ assessments. In the context of the SCT, it would be expected that improvements to behavior would be related to changes in knowledge. A pilot study that examined nutrition knowledge and VM score in college students detected a significant and moderate correlation between these at a single timepoint. When BMI was considered in this correlation as well, VM scores explained nearly 27% of the knowledge score variance [52]. The difference between findings in college students compared to the present study could be speculated to be due to the relatively little control children have over the foods available in the setting where they eat. Therefore, their food choices are often dependent on the foods purchased and/or prepared for them whereas college students have more autonomy with food choices.

At the onset of the present study, only a few studies had been published using the Veggie Meter^®^ [30] and none in this specific population. As a result, refinement of the data collection protocol was required in order to collect these data more efficiently. At the first data collection time points, students were not asked to clean their hands prior to measurements and the index finger on the dominant hand was used. After the publication of more research using the Veggie Meter^®^, the protocol evolved to include hand cleaning and the use of the non-dominant ring finger [42]. Since completion of this study, users of the Veggie Meter^®^ worked together to create a comprehensive set of recommendations for use in different research settings [53]. Future studies utilizing the Veggie Meter^®^ in this population should follow these guidelines to ensure consistency with other research. When considering the feasibility of using the Veggie Meter^®^ in school-based programs, being efficient with classroom time and minimizing how long students are away from learning is a critical consideration. With respect to other assessment methods that have been used to evaluate dietary intake, the Veggie Meter^®^ can be a minimally disruptive choice; especially if youth are excused from class individually or in small groups as the measurements themselves only take about 60 s each. Previous SHCP research used FFQs with lengthy introductions and instructions therefore making this likely a time saving method, comparatively [31].

This study has several limitations; key among these was the small sample size. Another noteworthy limitation is the interruption in study completion due to the COVID-19 pandemic. Because of the school closures, the final data collection timepoint did not occur. Despite this, available data suggest that the program resulted in increases in nutrition knowledge and consumption of carotenoid-containing fruits and vegetables. In particular, these data support the use of the Veggie Meter^®^ in future evaluations as an objective measure of change in fruit and vegetable consumption. In addition to a small sample size, data for students who did not complete all three assessments for a particular measure were excluded. This may have biased the results if the missing data are not randomly distributed. While significance was able to be achieved for some outcomes, the lack of statistical power may have impacted other outcomes. Another potential limitation is the changes to the VM administration protocol over the course of the study, with addition of hand cleaning after the first time point and the use of the non-dominant ring finger in the third timepoint. It is speculated that the addition of hand cleaning to the second and third timepoints would have resulted in lower scores than those observed, as hand cleaning removes residues that may artificially elevate VM scores, such as colored ink, or orange-colored powder from snack foods commonly consumed by this population. While recommendations for research protocol recommend the use of the non-dominant ring finger, a study of individuals of different skin pigmentations using different digits has yet to be published. Another limitation to consider is that the device may be cost prohibitive, with a one-time cost of approximately $15,000 per device. While this cost may be insurmountable for small programs, it can be more feasible as part of state level budgets or as part of large public health initiatives. One such example is the San Francisco Department of Public Health, which uses the Veggie Meter^®^ to assess the Healthy Apple Program [54,55]. In the specific case of this group, the cost of the device was split across departmental funding and state funding through CFHL, UC.

## 5. Conclusions

Overall, while this study was small, findings indicate that the Veggie Meter can be a useful tool for assessing dietary intake of fruits and vegetables in a low-income, school-based setting. Further, this study resulted in recommendations for a refined protocol for use of the Veggie Meter. By eliminating the time intensive, burdensome, and potentially biased methods of recall, a picture of fruit and vegetable intake can be elucidated using the objective and non-invasive technique. While more research should be conducted in the future, this study adds to the growing body of literature suggesting that the Veggie Meter^®^ is a valid and useful tool in this field.

## Figures and Tables

**Table 1:** Participant Characteristics at Baseline.

Characteristic	Percent (*n*)
Age	
9 years	97.0 (32)
10 years	3.0 (1)
Sex	
Female	51.4 (18)
Male	48.6 (17)
Race/ethnicity	
American Indian/Alaskan Native	2.9 (1)
Asian/Pacific Islander	20.0 (7)
Caucasian/white, not Hispanic origin	34.3 (12)
Latino/Hispanic	22.9 (8)
Other	2.9 (1)
Multiple Selected	14.3 (5)
No response	2.9 (1)
Household income	
$0–$19,000	5.7 (2)
$20,000–$39,999	31.4 (11)
$40,000–$59,999	17.1 (6)
$60,000–$79,999	11.4 (4)
$80,000–$99,999	5.7 (2)
$100,000 or more	22.9 (8)
Mother Education (*n* = 25)	
8th–11th	4.0 (1)
Finished high school or have a GED	8.0 (2)
Vocational/technical	4.0 (1)
Some college	48.0 (12)
Associate’s degree	12.0 (3)
Bachelor’s degree	16.0 (4)
Postgraduate	8.0 (2)
Father Education (*n* = 18)	
8th–11th grade	5.6 (1)
Finished high school or have a GED	16.7 (3)
Vocational/technical	11.1 (2)
Some college	33.3 (6)
Associate’s degree	5.6 (1)
Bachelor’s degree	22.2 (4)
Postgraduate	5.6 (1)
Other Primary Parent Education (*n* = 4)	
Finished high school or have a GED	25.0 (1)
Associate’s degree	25.0 (1)
Bachelor’s degree	25.0 (1)
Postgraduate	25.0 (1)
Other Secondary Parent Education (*n* = 4)	
8th or less	25.0 (1)
Some college	25.0 (1)
Associate’s degree	25.0 (1)
Bachelor’s degree	25.0 (1)
Smoker in Household	
Yes	8.6 (3)
No	82.9 (29)
No response	8.6 (3)

**Table 2:** Mean nutrition knowledge, BMI percentile-for-age, and VM score for each timepoint.

	Fall 2018 Mean (SD)	Spring 2019 Mean (SD)	Fall 2019 Mean (SD)	F	*p*
Nutrition Knowledge	9.28 (3.31) ^a^	10.52 (3.27) ^a,b^	11.09 (3.51) ^b^	5.51 (2, 48)	0.007
BMI Percentile	63.99 (30.11) ^a^	65.56 (29.48) ^a^	66.71 (29.88) ^a^	2.137 (2, 68)	0.126
VM Score	156.20 (78.03) ^a^	211.00 (76.50) ^b^	195.43 (64.10) ^b^	6.63 (2, 68)	0.002

Different letters indicate statistically significant differences with *p* < 0.05.

**Table 3:** Correlations between change in BMI percentile-for-age and change in VM score and VM score and knowledge.

	Correlation Coefficient	*p*-Value
Change in BMI Percentile-for-Age and Change in VM Score		
Fall 2018 to Spring 2019	0.157	0.354
Fall 2018 to Fall 2019	0.014	0.930
VM Score and Knowledge		
Fall 2018	0.269	0.034
Spring 2019	−0.018	0.912
Fall 2019	0.068	0.514

**Table 4:** Updates to protocol over course of study.

Time Point	Change to Protocol	Observation That Led to Change	Anticipated Impact
Spring 2019	Addition of hand-cleaning step using disposable hand sanitizing wipes	Presence of colored ink on some hands, which may artificially elevate VM score	Potential of reduced VM score due to elimination of pigments on hands due to snack foods or markers
Spring 2019	Addition of direction to create a line with removable tape for students to stand behind	Students would attempt to crowd around device, causing researchers to pause data collection repeatedly to ask students to move back	Potential to streamline data collection and reduce total time required
Fall 2019	Ring finger of non-dominant hand rather than index finger of dominant hand	Based on research suggesting ring finger of non-dominant hand [42]	Potential of reduced VM score due to variability of carotenoids in left versus right hands

## Data Availability

The data presented in this study may be provided on request from the corresponding author once IRB approval and appropriate data use agreements have been obtained.

## References

[1] Stierman B., Ogden C.L., Yanovski J., Martin C.B., Sarafrazi N., Hales C.M. (2021). Changes in adiposity among children and adolescents in the United States, 1999–2006 to 2011–2018. Am. J. Clin. Nutr..

[2] Liu J., Rehm C.D., Onopa J., Mozaffarian D. (2020). Trends in Diet Quality Among Youth in the United States, 1999–2016. JAMA.

[3] Sanyaolu A., Okorie C., Qi X., Locke J., Rehman S. (2019). Childhood and Adolescent Obesity in the United States: A Public Health Concern. Glob. Pediatr. Health.

[4] Thomson J.L., Tussing-Humphreys L.M., Goodman M., Landry A. (2019). Diet quality in a nationally representative sample of American children by sociodemographic characteristics. Am. J. Clin. Nutr..

[5] Wambogo E.A., Ansai N., Ahulwalia N., Ogden C.L. (2020). Fruit and Vegetable Consumption among Children and Adolescents in the United States, 2015–2018.

[6] Landry M.J., van den Berg A.E., Asigbee F.M., Vandyousefi S., Ghaddar R., Davis J.N. (2019). Child-Report of Food Insecurity Is Associated with Diet Quality in Children. Nutrients.

[7] Molitor F., Sugerman S., Yu H., Biehl M., Aydin M., Levy M., Ponce N.A. (2015). Reach of Supplemental Nutrition Assistance Program–Education (SNAP–Ed) Interventions and Nutrition and Physical Activity-Related Outcomes, California, 2011–2012. Prev. Chronic Dis..

[8] Rivera R.L., Maulding M.K., Eicher-Miller H. (2019). Effect of Supplemental Nutrition Assistance Program–Education (SNAP-Ed) on food security and dietary outcomes. Nutr. Rev..

[9] Naja-Riese A., Keller K., Bruno P., Foerster S.B., Puma J., Whetstone L., MkNelly B., Cullinen K., Jacobs L., Sugerman S. (2019). The SNAP-Ed Evaluation Framework: Demonstrating the impact of a national framework for obesity prevention in low-income populations. Transl. Behav. Med..

[10] Hayes D., Contento I.R., Weekly C. (2018). Position of the Academy of Nutrition and Dietetics, Society for Nutrition Education and Behavior, and School Nutrition Association: Comprehensive Nutrition Programs and Services in Schools. J. Acad. Nutr. Diet..

[11] Committee on Progress in Preventing Childhood Obesity (2007). Progress in Preventing Childhood Obesity: How Do We Measure Up?.

[12] Rochira A., Tedesco D., Ubiali A., Fantini M.P., Gori D. (2020). School gardening activities aimed at obesity prevention improve body mass index and waist circumference parameters in school-aged children: A systematic review and meta-analysis. Child Obes..

[13] Simmonds M., Llewellyn A., Owen C.G., Woolacott N. (2016). Predicting adult obesity from child-hood obesity: A systematic review and meta-analysis. Obes. Rev..

[14] Buscot M.-J., Thomson R.J., Juonala M., Sabin M.A., Burgner D.P., Lehtimäki T., Hutri-Kähönen N., Viikari J.S.A., Jokinen E., Tossavainen P. (2018). BMI Trajectories Associated With Resolution of Elevated Youth BMI and Incident Adult Obesity. Pediatrics.

[15] Tugault-Lafleur C.N., Black J.L., Barr S. (2017). A Systematic Review of Methods to Assess Children’s Diets in the School Context. Adv. Nutr..

[16] Nguyen L.M., Scherr R.E., Linnell J.D., Ermakov I.V., Gellermann W., Jahns L., Keen C.L., Miyamoto S., Steinberg F.M., Young H.M. (2015). Evaluating the relationship between plasma and skin carotenoids and reported dietary intake in elementary school children to assess fruit and vegetable intake. Arch. Biochem. Biophys..

[17] Blom-Hoffman J., Leff S.S., Franko D.L., Weinstein E., Beakley K., Power T.J. (2009). Consent Procedures and Participation Rates in School-Based Intervention and Prevention Research: Using a Multi-Component, Partnership-Based Approach to Recruit Participants. Sch. Ment. Health.

[18] Harnack L., Himes J.H., Anliker J., Clay T., Gittelsohn J., Jobe J.B., Ring K., Snyder P., Thompson J., Weber J.L. (2004). Intervention-related Bias in Reporting of Food Intake by Fifth-Grade Children Participating in an Obesity Prevention Study. Am. J. Epidemiol..

[19] Ruiz L.D., Zuelch M.L., Dimitratos S.M., Scherr R.E. (2019). Adolescent Obesity: Diet Quality, Psychosocial Health, and Cardiometabolic Risk Factors. Nutrients.

[20] Scherr R., Linnell J.D., Dharmar M., Beccarelli L.M., Bergman J.J., Briggs M., Brian K.M., Feenstra G., Hillhouse J.C., Keen C.L. (2017). A Multicomponent, School-Based Intervention, the Shaping Healthy Choices Program, Improves Nutrition-Related Outcomes. J. Nutr. Educ. Behav..

[21] Davis J.N., Ventura E.E., Cook L.T., Gyllenhammer L.E., Gatto N.M. (2011). LA Sprouts: A Gardening, Nutrition, and Cooking Intervention for Latino Youth Improves Diet and Reduces Obesity. J. Am. Diet. Assoc..

[22] Qi Y., Hamzah S.H., Gu E., Wang H., Xi Y., Sun M., Rong S., Lin Q. (2021). Is School Gardening Combined with Physical Activity Intervention Effective for Improving Childhood Obesity? A Systematic Review and Meta-Analysis. Nutrients.

[23] Hoelscher D.M., Springer A.E., Ranjit N., Perry C.L., Evans A.E., Stigler M., Kelder S.H. (2010). Reductions in Child Obesity Among Disadvantaged School Children With Community Involvement: The Travis County CATCH Trial. Obesity.

[24] Di Noia J., Gellermann W. (2021). Use of the Spectroscopy-Based Veggie Meter^®^ to Objectively Assess Fruit and Vegetable Intake in Low-Income Adults. Nutrients.

[25] Martinelli S., Acciai F., Tasevska N., Ohri-Vachaspati P. (2021). Using the Veggie Meter in Elementary Schools to Objectively Measure Fruit and Vegetable Intake: A Pilot Study. Methods Protoc..

[26] May K., Pitts S.J., Carraway-Stage V., Kelley C., Burkholder S., Fang X., Zeng A., Lazorick S. (2020). Use of the Veggie Meter^®^ as a tool to objectively approximate fruit and vegetable intake among youth for evaluation of preschool and school-based interventions. J. Hum. Nutr. Diet..

[27] Bakırcı-Taylor A.L., Reed D.B., McCool B., Dawson J.A. (2019). mHealth Improved Fruit and Vegetable Accessibility and Intake in Young Children. J. Nutr. Educ. Behav..

[28] Mayne S.T., Cartmel B., Scarmo S., Jahns L., Ermakov I.V., Gellermann W. (2013). Resonance Raman spectroscopic evaluation of skin carotenoids as a biomarker of carotenoid status for human studies. Arch. Biochem. Biophys..

[29] Ermakov I.V., Ermakova M., Sharifzadeh M., Gorusupudi A., Farnsworth K., Bernstein P.S., Stookey J., Evans J., Arana T., Tao-Lew L. (2018). Optical assessment of skin carotenoid status as a biomarker of vegetable and fruit intake. Arch. Biochem. Biophys..

[30] Pitts S.B.J., Jahns L., Wu Q., Moran N., Bell R., Truesdale K.P., Laska M.N. (2018). A non-invasive assessment of skin carotenoid status through reflection spectroscopy is a feasible, reliable and potentially valid measure of fruit and vegetable consumption in a diverse community sample. Public Health Nutr..

[31] Scherr R., Linnell J.D., Smith M.H., Briggs M., Bergman J., Brian K.M., Dharmar M., Feenstra G., Hillhouse C., Keen C.L. (2014). The Shaping Healthy Choices Program: Design and Implementation Methodologies for a Multicomponent, School-Based Nutrition Education Intervention. J. Nutr. Educ. Behav..

[32] Taylor J.C., Zidenberg-Cherr S., Linnell J.D., Feenstra G., Scherr R.E. (2017). Impact of a multicomponent, school-based nutrition intervention on students’ lunchtime fruit and vegetable availability and intake: A pilot study evaluating the Shaping Healthy Choices Program. J. Hunger Environ. Nutr..

[33] Linnell J.D., Zidenberg-Cherr S., Briggs M., Scherr R., Brian K.M., Hillhouse C., Smith M.H. (2016). Using a Systematic Approach and Theoretical Framework to Design a Curriculum for the Shaping Healthy Choices Program. J. Nutr. Educ. Behav..

[34] Fetter D.S., Scherr R.E., Linnell J.D., Dharmar M., Schaefer S.E., Zidenberg-Cherr S. (2018). Effect of the Shaping Healthy Choices Program, a Multicomponent, School-Based Nutrition Intervention, on Physical Activity Intensity. J. Am. Coll. Nutr..

[35] Bergman J., Linnell J.D., Scherr R.E., Ginsburg D.C., Brian K.M., Carter R., Donohue S.S., Klisch S., Lawry-Hall S., Pressman J. (2018). Feasibility of Implementing a School Nutrition Intervention That Addresses Policies, Systems, and Environment. J. Ext..

[36] Fetter D.S., Dharmar M., Lawry-Hall S., Pressman J., Chapman J., Scherr R.E. (2019). The Influence of Gain-Framed and Loss-Framed Health Messages on Nutrition and Physical Activity Knowledge. Glob. Pediatr. Health.

[37] Scherr R.E., Jones A.M., Colorafi R., Klisch S., Linnell J.D., Soule K.E. (2020). Assessing the Effectiveness of an Extender Model Partnership in Implementing a Multicomponent, School-Based Nutrition Intervention. Health Promot. Pract..

[38] Morris J.L., Zidenberg-Cherr S. (2001). Nutrition to Grow On: A Garden-Enhanced Nutrition Education Curriculum for Upper Elementary School Children.

[39] Burrows T.L., Warren J.M., Colyvas K., Garg M.L., Collins C.E. (2009). Validation of Overweight Children’s Fruit and Vegetable Intake Using Plasma Carotenoids. Obesity.

[40] Kuczmarski R.J., Ogden C.L., Guo S.S., Grummer-Strawn L.M., Flegal K.M., Mei Z., Wei R., Curtin L.R., Roche A.F., Johnson C.L. (2002). 2000 CDC Growth Charts for the United States: Methods and Development.

[41] Barlow S.E. (2007). Expert Committee Recommendations Regarding the Prevention, Assessment, and Treatment of Child and Adolescent Overweight and Obesity: Summary Report. Pediatrics.

[42] Ansu V., Dickinson S., Fly A. (2019). Digit Variability in Carotenoid Scores Obtained with the Veggie Meter: A Pilot Study (P02-001-19). Curr. Dev. Nutr..

[43] Radtke M.D., Pitts S.J., Jahns L., Firnhaber G.C., Loofbourrow B.M., Zeng A., Scherr R. (2020). Criterion-Related Validity of Spectroscopy-Based Skin Carotenoid Measurements as a Proxy for Fruit and Vegetable Intake: A Systematic Review. Adv. Nutr..

[44] Collins C., Watson J., Burrows T. (2010). Measuring dietary intake in children and adolescents in the context of overweight and obesity. Int. J. Obes..

[45] Kroes R., Müller D., Lambe J., Löwik M., van Klaveren J., Kleiner J., Massey R., Mayer S., Urieta I., Verger P. (2002). Assessment of intake from the diet. Food Chem. Toxicol..

[46] Natarajan L., Flatt S.W., Sun X., Gamst A.C., Major J.M., Rock C.L., Al-Delaimy W., Thomson C.A., Newman V.A., Pierce J.P. (2006). Validity and systematic error in measuring carotenoid consumption with dietary self-report instruments. Am. J. Epidemiol..

[47] Shim J.-S., Oh K., Kim H.C. (2014). Dietary assessment methods in epidemiologic studies. Epidemiol. Health.

[48] U.S. Department of Agriculture, Food and Nutrition Service (2016). The Supplemental Nutrition Assistance Program Education (SNAP-Ed) Evaluation Framework: Nutrition, Physical Activity, and Obesity Prevention Indicators: Interpretive Guide to the SNAP-Ed Evaluation Framework.

[49] Puma J.E., Young M., Foerster S., Keller K., Bruno P., Franck K., Naja-Riese A. (2021). The SNAP-Ed Evaluation Framework: Nationwide Uptake and Implications for Nutrition Education Practice, Policy, and Research. J. Nutr. Educ. Behav..

[50] SNAP-Ed Funding Allocations. https://snaped.fns.usda.gov/program-administration/snap-ed-funding-allocations.

[51] Bandura A. (1986). Social Foundations of Thought and Action: A Social Cognitive Theory.

[52] Jones A., Radtke M., Chodur G., Scherr R. (2020). Assessing the Relationship Between Nutrition Knowledge and Skin Carotenoids in University Students. Curr. Dev. Nutr..

[53] Radtke M.D., Poe M., Stookey J., Pitts S.J., Moran N., Landry M.J., Rubin L.P., Stage V.C., Scherr R. (2021). Recommendations for the Use of the Veggie Meter^®^ for Spectroscopy-Based Skin Carotenoid Measurements in the Research Setting. Curr. Dev. Nutr..

[54] Stookey J., Evans J., Chan C., Tao-Lew L., Arana T., Arthur S. (2017). Healthy apple program to support child care centers to alter nutrition and physical activity practices and improve child weight: A cluster randomized trial. BMC Public Health.

[55] San Francisco Department of Public Health Website Maternal, Child & Adolescent Health. https://www.sfdph.org/dph/comupg/oprograms/MCH/Epi.asp.

